# On the Feasibility of Utilizing Allogeneic Bone Blocks for Atrophic Maxillary Augmentation

**DOI:** 10.1155/2014/814578

**Published:** 2014-09-11

**Authors:** Alberto Monje, Michael A. Pikos, Hsun-Liang Chan, Fernando Suarez, Jordi Gargallo-Albiol, Federico Hernández-Alfaro, Pablo Galindo-Moreno, Hom-Lay Wang

**Affiliations:** ^1^Department of Periodontics and Oral Medicine, School of Dentistry, University of Michigan, Ann Arbor, MI, USA; ^2^Private practice, Palm Beach, FL, USA; ^3^Department of Oral Surgery and Implant Dentistry, International University of Catalonia, Barcelona, Spain; ^4^Department of Oral Surgery and Implant Dentistry, University of Granada, Granada, Spain

## Abstract

*Purpose*. This systematic review was aimed at assessing the feasibility by means of survival rate, histologic analysis, and causes of failure of allogeneic block grafts for augmenting the atrophic maxilla.* Material and Methods*. A literature search was conducted by one reviewer in several databases. Articles were included in this systematic review if they were human clinical trials in which outcomes of allogeneic bone block grafts were studied by means of survival rate. In addition other factors were extracted in order to assess their influence upon graft failure.* Results*. Fifteen articles fulfilled the inclusion criteria and subsequently were analyzed in this systematic review. A total of 361 block grafts could be followed 4 to 9 months after the surgery, of which 9 (2.4%) failed within 1 month to 2 months after the surgery. Additionally, a weighed mean 4.79 mm (95% CI: 4.51–5.08) horizontal bone gain was computed from 119 grafted sites in 5 studies. Regarding implant cumulative survival rate, the weighed mean was 96.9% (95% CI: 92.8–98.7%), computed from 228 implants over a mean follow-up period of 23.9 months. Histologic analysis showed that allogeneic block grafts behave differently in the early stages of healing when compared to autogenous block grafts.* Conclusion*. Atrophied maxillary reconstruction with allogeneic bone block grafts represents a reliable option as shown by low block graft failure rate, minimal resorption, and high implant survival rate.

## 1. Introduction

An unavoidable series of events results in bone resorption after tooth extraction [1–4]. Consequently, grafting procedures are common treatments in the dental setting to correct these deficiencies and to allow for proper three-dimensional implant placement. Numerous alternatives such as distraction osteogenesis or guided bone regeneration (GBR) have been proposed [5]. Recently, advances in implant macrodesign [6–8] as well as technical advancement [9–11] have limited the need for grafting procedures. Nonetheless, for extensive/severely atrophic maxillary ridges, block grafting remains a predictable approach [12, 13].

For block grafting procedures, the use of autogenous bone has been claimed to be the “gold standard” due to its osteogenic, osteoinductive, and osteoconductive properties [14]. While intraoral bone block grafts such as mandibular ramus and symphysis grafts can be harvested with minimal morbidity the amount of available bone remains its big disadvantage. On the other hand, extraoral bone block grafts, such as calvaria or iliac crest, provide the greater quantity of bone but increased cost and are often associated with high morbidity in the donor site. Due to these limitations and drawbacks, clinicians have opted to use either allogeneic or xenogeneic bone blocks for the reconstruction of severe atrophy defects of the maxilla [15–31]. When these alternatives are employed, they not only reduce the possibility of morbidity, but also shorten the treatment length, hence increasing patients' acceptance and satisfaction.

Nevertheless, integration of allogeneic or xenogeneic block bone to the native bone might be arduous due to the scarcity of cells within the graft. The mechanism of forming new mineralized tissue is mediated by the mesenchymal cells that can differentiate into osteoblasts which are coordinated by glycoproteins [32]. Following an inflammatory process, new bone is formed after gradual substitution [33] which leads to obtain implant primary stability and subsequent osseointegration.

Promising results have been reported with regards to the use these alternative block grafts plus different biomaterials for bone regeneration [35, 36]. Depending on their sources, they can be obtained either from human cadaver (allogeneic grafts) or from animal origin (xenogeneic grafts). However, the fate of xenografts remains unclear due to their nonosteoinductive capacity. On the other hand, the use of allogeneic block graft harvested from the same species represents a better alternative to the use of autogenous block bone. The first bone allografts were performed in late 19th century by a group of surgeons whom reconstructed an infected humerus with a graft harvested from the tibia of the same patient [37]. In 1990 the US Navy Tissue Bank was established which made the use of bone allografts popular [38]. In 1999, the first case of allogeneic block bone graft for regeneration in oral surgery was reported. In that case, dental implants for oral rehabilitation were successfully placed 3 months after the grafting procedure [36]. Since then, many studies have been carried out intending to show the reliability of allografts to assist in bone regeneration. Nonetheless, as far as we know, there is limited information that has been pooled and analyzed in an attempt to answer the fate of allogeneic block grafts for the rehabilitation of atrophic maxillae [15–21, 24–31]. Therefore, this systematic review aimed at assessing the feasibility of allogeneic block grafts by means of survival rate, histologic analysis, and causes of failure, for augmentation of the atrophic maxilla.

## 2. Material and Methods 

### 2.1. Information Sources and Development of Focused Question

An electronic literature search was conducted by one reviewer (AM) in several databases, including MEDLINE, EMBASE, Cochrane Central Register of Controlled Trials, and Cochrane Oral Health Group Trials Register databases for articles written in English from January, 2000, up to December, 2013. The PICO question was as follows. Do edentulous patients restored by allograft bone blocks in the atrophic maxillae have acceptable clinical outcomes when compared to other types of block grafts by means of survival rate and histologic examination? The reporting of these meta-analyses adhered to the PRISMA (Preferred Reporting Items for Systematic Review and Meta-Analyses) statement [39].

### 2.2. Screening Process

Combinations of controlled terms (MeSH and EMTREE) and keywords were used whenever possible. The search terms used, where “[mh]” represented the MeSH terms and “[tiab]” represented title and/or abstract, for the PubMed search were “bone graft” [mh] OR “bone grafting” [ti] OR “dental implantation, endosseous” [mh] OR “dental implants” [mh] AND “bone graft” [tiab] OR “grafting” [mh] AND block [tiab] AND allogeneic [tiab] English [la] NOT letter [pt] OR comment [pt] OR editorial [pt] NOT “animals” [mh] NOT “humans” [mh]. Additionally, a manual search of implant-related journals, including* Clinical Implant Dentistry and Related Research, Journal of Oral and Maxillofacial Implants, Clinical Oral Implants Research, Implant Dentistry, Journal of Dental Research, Journal of Clinical Periodontology, Journal of Periodontology, and The International Journal of Periodontics & Restorative Dentistry, *from January, 2012, up to December, 2013, was also performed to ensure a thorough screening process.

### 2.3. Eligibility Criteria

Articles were included in this systematic review if they met the following inclusion criteria: prospective human clinical trials in which outcomes of allograft bone blocks were studied by means of survival rate. Accordingly, several factors such as study design, number of patients included at the last follow-up assessment, number of sites grafted, type of bone augmentation (vertical/horizontal/both), type of bone block studied, placement of membrane, whether any other grafting material was further used, and healing period were extracted from the selected studies and analyzed. Furthermore, in order to address the aim of this study, other parameters related to block graft survival, block graft behavior (resorption pattern), and histologic findings were further extracted (Table 1). On the contrary, case report or case series with less than 10 subjects included, systematic reviews, animal studies, retrospective cohort, and those studies in which information was not clear enough were excluded from this meta-analysis. References in the excluded articles were also checked seeking for studies that fulfilled our inclusion criteria. The Newcastle-Ottawa scale (NOS) was used to assess the quality of such studies for a proper understanding of nonrandomized studies [40].

### 2.4. Data Analysis

Demographic data, graft features, and surgical techniques were extracted from individual study. For meta-analyses of the horizontal bone gain and implant survival rate, the numbers of blocks and implants and the mean horizontal bone gain with standard deviation as well as the mean implant survival rate were retrieved from the included studies, if available. The weighted mean (WM) and the 95% confidence interval (CI) of the two variables were estimated using a computer program (Comprehensive Meta-analysis Software, Biostat, NJ, USA). The random effect model was applied to account for methodological differences among studies. Forest plots were computed to graphically represent the weighed means and 95% CI of the outcomes using “block graft site” and “implant” as the analysis unit for the horizontal bone gain and implant survival, respectively. For block survival rate, the Kaplan-Meier estimator was used to plot the survival curve. The number of grafts, mean followup time, the number of failed grafts, and time when the grafts failed were extracted from the studies. Data were input into a spreadsheet and computed by commercially available software (SPSS v 22.0, IBM, Chicago, IL, USA). All analyses were performed by one blinded investigator (H-LC).

## 3. Results

### 3.1. Study Selection

**Figure 1:**
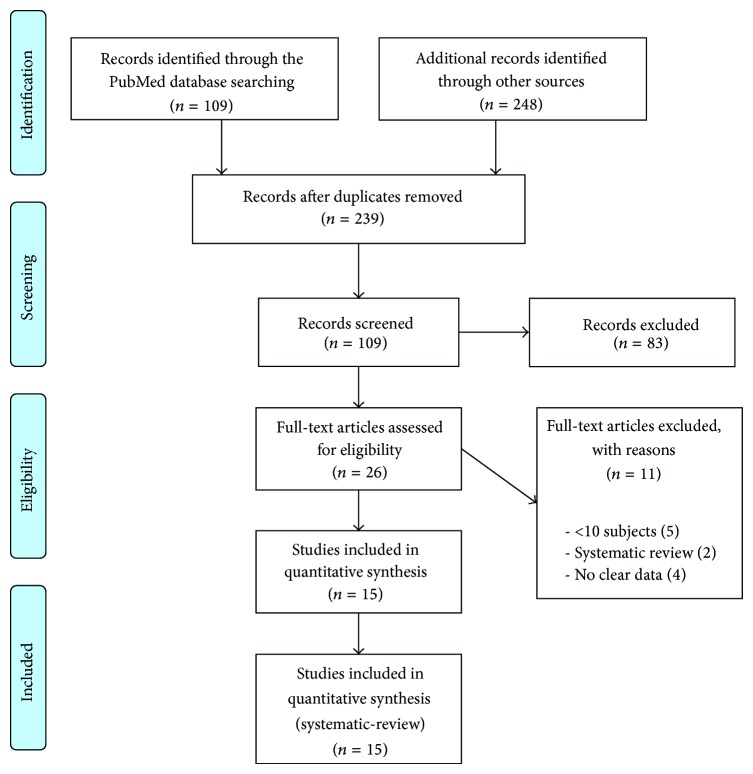
Identification, screening, and eligibility criteria for the studies included in this systematic review.

An initial screening yielded a total of 239 articles, of which 109 potentially relevant articles were selected after evaluation of their abstract. Next, 26 papers of full text of these articles were then obtained and reviewed. Of these, only 15 articles fulfilled the inclusion criteria and subsequently were analyzed in this systematic review (Figure 1). Details of all included studies were summarized in Table 1. Reasons for exclusion were case reports or <10 subjects included (5) [22, 41–44], and systematic/narrative reviews (2) [35, 36]. In addition, four more studies were excluded due to not clearly displaying appropriate data or to providing lack of the required data for this systematic review [23, 34, 45, 46]. On the other hand, all the included studies detected were prospective case series (14) and randomized controlled trials (1) [15–21, 24–31]. In some instances, when there was possibility to clearly identify blocks survival/failure by location, mandible block grafts were excluded inasmuch as the aim of the study was only to report their feasibility in the maxillae.

### 3.2. Study Quality

All the articles included in the present systematic review were prospective human clinical trials evaluating survival of allogeneic block grafts placed in the atrophic maxilla. The Newcastle-Ottawa scale (NOS) was used to assess the quality of such studies for a proper understanding of nonrandomized studies [40]. The fact that some studies came from the same group might leads to risk of bias due to repeated data; however, it was thoroughly assessed to make sure this was not the case. Thereupon, according to the NOS, a mean score of 6.06 ± 1.04 was obtained, indicating the adequate (medium-high) level of evidence of the included studies.

### 3.3. Failure Rate of Allogeneic Bone Blocks

**Figure 2:**
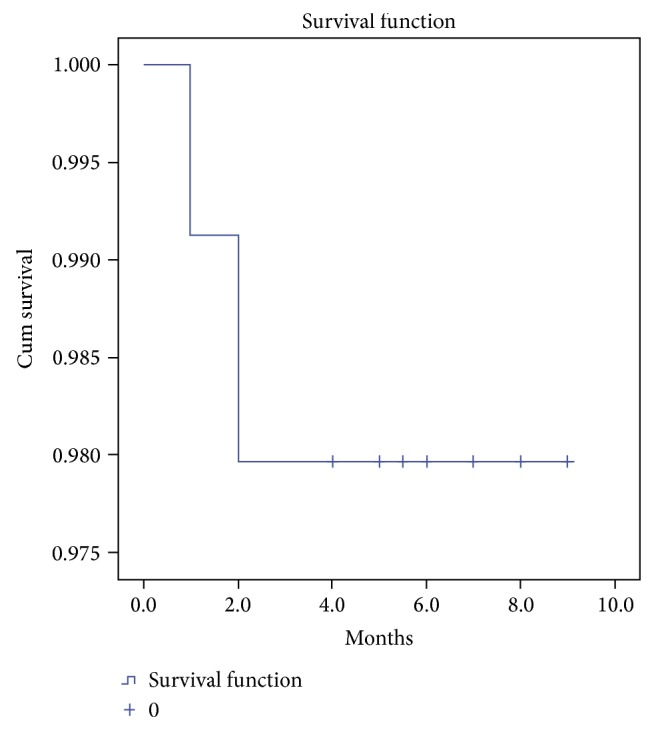
Cumulative survival rate of allogeneic bone block grafts placed in the maxillae.

A total of 361 block grafts were followed until 4 to 9 months after the surgery, of which 9 failed within 1 to 2 months after the surgery. The cumulative survival rate of the block grafts was 98% (Figure 2). Of the 9 reported failed cases, 5 were corticocancellous and the other 4 were cancellous grafts; 7 were combined with the use of membrane and the other 2 were not. Due to the limited number of failed cases, the effect of the graft type and membrane use on graft failure was not analyzed.

### 3.4. Timing and Causes of Failure of Allogeneic Bone Blocks

It was shown that block grafts failed generally in early stages of graft healing (≤2 months) [16, 20, 27, 30]. This suggested that the odds of grafts success increase from the third month on. Early membrane exposure was found to be the main reason for block graft failure [16, 20, 27, 30]. Moreover, it was reported that fixation screw loosening was the second leading cause for block graft failure [29].

### 3.5. Resorptive Pattern and Final Bone Gain of Allogeneic Bone Blocks

**Figure 3:**
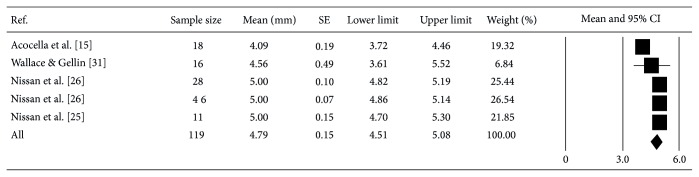
A weighed mean 4.79 mm (95% CI: 4.51–5.08) horizontal bone gain was computed from 119 grafted sites in 5 studies.

A weighed mean of 4.79 mm (95% CI: 4.51–5.08) horizontal bone gain was computed from 119 grafted sites in 5 studies [15, 24–26, 31]. Allogeneic block graft resorption ranged from 10 ± 10% [24] to 52 ± 25.97% [21] at 6 months after grafting (Figure 3). However, it is important to note that the mean value was found to be relatively low (21.70 ± 30.55%) [15, 20, 21, 24]. In addition, high heterogeneity was also found among these studies. Interestingly, even though the sample size is small it was noticed the longer the healing, the less bone gain was obtained. On the other hand, allogeneic block grafts resulted in 2 ± 0.5 mm vertical bone augmentation [24, 26].

### 3.6. Implant Cumulative Survival Rate

**Figure 4:**
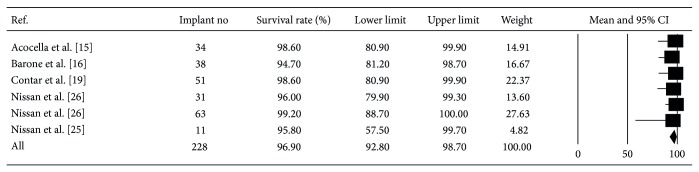
The weighed mean implant survival rate was 96.9% (95% CI: 92.8–98.7%), computed from 228 implants over a mean follow-up period of 23.9 months.

The weighed mean implant survival rate was 96.9% (95% CI: 92.8–98.7%), computed from 228 implants over a mean follow-up period of 23.9 months (Figure 4) [15, 16, 19, 20, 24–27].

### 3.7. Histomorphometric and Histologic Characteristics of Allogeneic Bone Blocks

Six studies reported the histologic characteristics at reentry for implant placement [15, 18–21, 30]. Of these, only two compared the outcome with a control group, which in these cases were autogenous block grafts harvested from the mandibular ramus (Table 1) [21, 30]. Acocella et al. [15] showed that after a healing period of 9 months, a high number of empty osteocyte lacunae were still present. Additionally, newly formed bone (61.96 ± 11.77%) was surrounded by nonvital bone with empty osteocyte lacunae. Contar et al. [18] reported lamellar arrangement around Haversian canals interspersed with osteocytes in lacunae. In addition, in the center of the block grafts osteocytes with higher number of empty lacunae were noticed. On the other hand, when histologic results are compared among groups, behavioral dissimilarities are displayed. Lumetti et al. [21] demonstrated that after 6 months of healing osteocyte lacunae were mostly empty for the allogeneic block graft group. Furthermore, it was reported that newly formed bone contained viable osteocytes at that point. In these samples, bone forming osteoblasts and fluorescent labeling were detected. Dense connective tissue with the presence of inflammatory cells and eroded areas were also observed in such group. Minimal differences were shown for the autogenous block grafts group in which no connective tissue was found and where the presence of inflammatory cells was meaningfully lower. Contrarily, Spin-Neto et al. [30] found major dissimilarities between the groups. For the allogeneic bone block large segments of necrotic bone with empty osteocytes lacunae and little osteoclastic activity, along with blood vessels invading the Haversian canals of the material were found. In addition, no direct contact between remodeled and grafted bone was found. For the autogenous block grafts small areas of necrotic bone with abundant presence of osteocytes were detected. Finally, no difference between the graft and the host bone was noticed.

## 4. Discussion

The use of autogenous grafts for bone augmentation of the atrophic maxilla was first documented by Branemark and is still considered the “gold standard” material due to their osteogenic potential for tissue regeneration [47]. Indeed, this property provides autogenous grafts more predictability by means of host-graft tissue integration. Nevertheless, it also presents some limitations. For instance, Nkenke et al. reported that patients might notice disturbances of the inferior alveolar nerve even 12 months after harvesting bone from the symphysis [48]. In addition, Clavero and Lundgren [49] found that half of the patients enrolled that underwent harvesting surgery from the mandibular ramus or chin experienced permanent altered sensation of the lower lip-chin. Other drawbacks are the additional cost and the possible need of general anesthesia and/or hospitalization. Also, excessive graft resorption of the autogenous bone block can be another concern. Nyström et al. observed a reduction in width of iliac crest onlay block grafts from 12.2 mm to 8.7 mm at 12 months [50]. Widmark et al. discovered that bone resorption of block grafts harvested from the mandible and used for horizontal augmentation of the anterior maxilla was 60% [51]. Similar findings were reported by Ozaki and Buchman in an animal study (56% of resorption of intramembranous blocks) [52]. Hence, all these facts have encouraged clinicians in seeking alternatives to autogenous bone for vertical and horizontal bone augmentation.

On the other hand, allogeneic grafts have proven to be successful in terms of integration with the host bone due to their osteoinductive potential [53, 54]. In addition, these grafts offer several benefits in comparison to autogenous grafts by means of reducing morbidity, discomfort, and operation time. Within limitations this systematic review showed that, regardless of subtype, allogeneic bone block grafts represent a feasible alternative to autogenous block grafts in augmenting the atrophic maxilla. Additionally, our results also confirm that allogeneic block grafts remain stable over the studies period when compared to previous findings [50–52]. Data from studies showed allogeneic block grafts resorbed ranged from 10 ± 10% [24] to 52 ± 25.97% [21] at 6 months after grafting. Nonetheless, it is important to note that the mean value was found to be relatively low (21.70 ± 30.55%) [15, 20, 21, 24], which is significantly lower than what Lumetti et al. [21], reported when fresh-frozen allogeneic block grafts were used.

Results from this review showed a mean gain of 4.79 mm horizontal and 2 mm vertical bone was obtained [15, 24–26, 31]. This is comparable to autogenous bone grafts but without the associated donor site morbidity and higher resorptive rate; hence, we can imply that allogeneic block grafts can be a good alternative graft material for augmenting atrophic maxilla. Even though our purpose was to assess the reliability of allogeneic block grafts to augment the atrophic maxilla vertically and horizontally, no clear conclusion can be drawn with regard to vertical bone augmentation due to the limited data. Rocchietta et al. point out that vertical bone augmentation represents a technical challenge and there is paucity of evidence to claim any treatment approach as the most predictable [55]. On the contrary, Nissan et al. [24, 26] showed that it is possible not only to succeed by means of stability but also to achieve nonnegligible bone gain of 2 ± 0.5 mm. Therefore, precautions must be exercised when interpreting the results obtained in this systematic review especially in the arena of vertical bone augmentation.

In order to accomplish the principle of GBR as described by Melcher [56], a membrane must be placed to cover the graft to exclude unwanted cells into the wound. Nonetheless, Kusiak et al. found that barrier membrane has a limited effect on the onlay block [57]. Interestingly, other authors claim that the use of membranes might lead to a higher prevalence of complications, such as membrane exposure and subsequent infection [51, 58]. Notwithstanding, by using newly developed bioabsorbable membranes, clinicians have achieved better results overcoming the drawbacks presented by the non-bioabsorbable membranes [59, 60]. In the present study, meta-analysis of the data becomes impossible due to number of failed cases. Nevertheless, only two out of the nine failed blocks membranes were not placed.

It is important to evaluate the survival rate of implants placed following ridge augmentation. Data from this systematic review showed a mean implant survival rate of 96.9% (95% CI: 92.8–98.7%), computed from 228 implants over a mean follow-up period of 23.9 months. Hence, it can be concluded that allogeneic block grafts for augmentation of resorbed maxillae behave similar to native bone in supporting implant osseointegration. This is in agreement with Clementini et al. who demonstrated a high survival rate as long as implants are placed following a delayed placement protocol after onlay bone grafting [61]. Nonetheless, the ideal time to place implants after allogeneic block grafting remains to be determined.

Another factor of importance is the histological behavior of allogeneic block grafts and their incorporation to host bone. Graft revascularization is critical to the success of bone grafting in general and to block bone grafting in particular. Allogeneic grafts, in contrast to xenogeneic grafts, still maintain vital cells despite the preservation process that they undergo [62]. Simpson et al. [62] in an* in vitro* study showed the osteopromotive capacity of fresh frozen allografts. This systematic review demonstrated that allogeneic block grafts in the early stages of healing behave differently than do autogenous block grafts. However it remains unclear about the fate of this biomaterial in the late stage of bone remodeling. Furthermore, it is worth noting that a high heterogeneity among studies existed when examining the histologic characteristics. While Lumetti et al. [21] reported minimal differences for allogeneic blocks when compared to autogenous blocks, Spin-Neto et al. [30] found major dissimilarities between them For the allogeneic bone block, large segments of necrotic bone with empty osteocytes lacunae and little osteoclastic activity, and minimal number of blood vessels invading Haversian canals were found. In addition, there is no direct contact between remodeled and grafted bone was found. For autogenous block grafts small areas of necrotic bone with abundant presence of osteocytes were detected. No difference between the graft and host bone was noticed [30].

Future research must be conducted to clarify numerous unknowns. From the clinical perspective, a large randomized clinical trial should be designed to compare the long-term fate of allogeneic blocks when compared to intramembranous and endochondral autogenous block grafts. In addition, it remains unclear which type of allogeneic block graft represents the most reliable one by means of bone gain and interaction with host bone. Generally speaking, bone resorption potentially relies upon numerous parameters that were shown to play a role; for instance, buccal bone thickness is known to determine the percentage of bone loss. Nevertheless, it is yet to be determined the influence of thickness upon final volume gain. Finally, it will be interesting to find out if additional of biologic agents (e.g., bone morphogenetic proteins) can be used to speed up or improve allogeneic block graft maturation.

## 5. Conclusion

Within the limitations of this systematic review, it can be concluded that the use of allogeneic bone block grafts represent a reliable alternative to autogenous block grafts for augmenting the atrophic maxilla. Furthermore, implants placed in allogeneic block augmented bone can achieve similar implant survival rates. However, due to the heterogeneity among the selected studies and limitation of sample size, results from this study should be interpreted with caution. Future studies to include larger sample size, longer followup, and better controlled are encouraged.

## Figures and Tables

**Table 1a:** (a)

Author (year)	Study design	Groups	Number of patients	Number of sites grafted	Location of grafted sites	Bone agumentation (V/H)	Type of bone block graft	Membrane (Y/N)	Additional grafting material/growth factor	Bone augmentation achieved at baseline	Healing period (months)	Resorption (%)
Acocella et al., (2012) [15]	Prospective case series	NCG	16	18	Anterior/posterior	H	Monocortical fresh-frozen	N	N	4.62 ± 0.8 mm	9	11.45 ± 8.37

Barone et al., (2009) [16]	Prospective case series	NCG	13	24	Anterior (13)/posterior (9)	H (19)/V (5)	Corticocancellous deep-frozen	N	Cancellous allograft particles	NM	5	NM

Chaushu et al., (2010) [17]	Prospective case series	NCG	101	90	Anterior (58)/posterior (32)	NC	Cancellous fresh-frozen	Y	N	NM	6	NM

Contar et al., (2009) [19]	Prospective case series	NCG	15	34	Anterior/posterior	H	Cancellous/cortical fresh-frozen	N	N	NM	NC	NM

Contar et al., (2011) [18]	Prospective case series	NCG	18	39	Anterior/posterior	NC	Cancellous/cortical fresh-frozen	N	N	NM	9	NM
							Cortical fresh-frozen					

Wallace and Gellin (2010) [31]	Prospective case series	NCG	12	16	Anterior/posterior	H	Cancellous fresh-frozen	Y	MCA + rhPDGF-BB	4.6 ± 5.2 mm	5	NM

Spin-Neto et al. (2013) [29]	Prospective case series	AL	13	17	Anterior (14)/posterior (3)	H	Corticocancellous deep-frozen	Y	N	NC	6	NC
		AT	13	17			Mandibular ramus					

Novell et al., (2012) [27]	Prospective case series	NCG	12	20	Anterior/posterior	H/H + V	Cortical/cancellous fresh-frozen	Y	Freeze-dried allograft particles	NC	NM	NM

Deluiz et al., (2013) [20]	Prospective case series	NCG	24	24	Anterior/posterior	H	Corticocancellous fresh-frozen	N	Freeze-dried allograft particles	NC	8	13.02 ± 3.86

Nissan et al., (2011) [26]	Prospective case series	NCG	20	28	Anterior	H (27)/V (12)	Cancellous fresh-frozen	Y	Particulate BBM	NM	6	NM

Nissan et al. (2011) [24]	Prospective case series	NCG	31	46	Anterior	H (42)/V (27)	Cancellous fresh-frozen	Y	Particulate BBM	NM	6	10 ± 1

Nissan et al., (2008) [25]	Prospective case series	NCG	11	11	Anterior	H/V	Cancellous fresh-frozen	Y	Particulate BBM	NM	6	NM

Lumetti et al., (2014) [21]	RCT	AL	12	12	Anterior/posterior	H	Corticocancellous fresh-frozen	Y	Particulate fresh-frozen	1.5 ± 0.91 cm^3^	6	52 ± 25.87
		AT	12	12			Mandibular ramus			0.44 ± 1.04 cm^3^		25 ± 12.73

Spin-Neto et al. (2013) [30]	Prospective case series	AL	6	17	Anterior/posterior	H	Cortical fresh-frozen	Y	N	NM	7	NM
		AT	6	12			Mandibular ramus			NM		NM

Peleg et al., (2010) [28]	Prospective case series	NCG	34	38	Anterior (31)/posterior (7)	H/H + V	Corticocancellous fresh-frozen	Y	N	NM	4	NM

**Table 1b:** (b)

Author (year)	Final bone gain (mm)	Number of implant placed	Implant loading protocol	Followup of implants (months)	Implant survival	Failed blocks (%)			Histological findings		
						Failed blocks (%)	Timing (months)	Cause	Timepoint (months)	Newly formed bone (%)	Characteristics
Acocella et al., (2012) [15]	4.09 ± 0.8	34	4	30	100	0	—	—	9	61.96 ± 11.77	A high number of empty osteocyte lacunae were still present and, fibrous tissue was more present than in the samples taken previously. Newly-formed bone was surrounded by non-vital bone with empty osteocyte lacunae in way of resorption

Barone et al., (2009) [16]	NM	38	NM	6	94.73	8.33	1	Early exposure and infection of vertical onlay grafts	NM	NM	NM

Chaushu et al., (2010) [17]	NM	NM	NM	NM	NM	6.66	NC	Membrane exposure, incision line opening, soft tissue perforations, recipient site infection	NM	NM	NM

Contar et al., (2009) [19]	NM	51	NC	35	100	0	—	—	NC	NM	Mature and compact osseous tissue surrounded by marrow spaces

Contar et al., (2011) [18]	NM	58	NM	NM	NM	0	—	—	9	NM	Lamellar arrangement around Haversian canals interspersed with osteocytes in lacunae. No evidence of inflammatory infiltrate. The central portions revealed osteocytes with higher number of empty lacunae

Wallace & Gellin (2010) [31]	8.39 ± 1.95	NM	NM	NM	NM	0	—	—	NM	NM	NM

Spin-Neto et al. (2013) [29]	NC	NM	NM	NM	NM	11.76	2	Fixation screws loosened causing inflammation	NM	NM	NM
		NM	NM	NM	NM	0	—	—			

Novell et al., (2012) [27]	NM	NC	NC	60	100	5	1	Failure occurred in the posterior area	NM	NM	NM

Deluiz et al., (2013) [20]	NC	75	NM	NM	98.67	0	—	—	4, 6, 8	NM	Newly formed bone with osteocytes observed in all the timepoints. Osteocyte presence was higher at 4 months. Vessels were also detected abundantly in the samples

Nissan et al., (2011) [24]	H (5 ± 0.5)/V (2 ± 0.5)	31	0 (12)/6 (19)	42	96	7.2	1	Because of soft tissue breakdown, infection and loss of fixation	NM	NM	NM

Nissan et al. (2011) [24]	H (5 ± 0.5)/V (2 ± 0.5)	63	6	34	100	4.4	1	Because of soft tissue breakdown, infection and loss of fixation	NM	NM	NM

Nissan et al., (2008) [25]	H (5 ± 0.5)/V (NM)	11	0	18	100	0	—	—	NM	NM	NM

Lumetti et al., (2014) [21]	NC	NM	NM	NM	NM	0	—	—	6	NC	Osteocyte lacunae mostly empty. Newly formed bone contained viable osteocytes. Bone forming osteoblasts and fluorescent labeling detected. Dense connective tissue with the presence of inflammatory cells (WM score = 1.67) and eroded areas
						0	—	—		NC	Osteocyte lacunae mostly empty. Newly formed bone contained viable osteocytes. Bone forming osteoblasts and fluorescent labeling detected. WM inflammatory score = 1

Spin-Neto et al. (2013) [29]	NM	40	NM	NM	NM	0	—	—	7	NM	Large segments of necrotic bone with empty osteocytes lacunae and little osteoclastic activity. Blood vessels were invading the Haversian canals of the material. No direct contact was found between remodeled and grafted bone. Some osteoclastic activity surrounded by connective tissue with no presence of inflammatory cells by newly formed bone failed to invade the graft.
	NM					0	—	—		NM	Small areas of necrotic bone with abundant presence of osteocytes. Inexistent difference between the grafted and the host bone

Peleg et al., (2010) [28]	NM	NC	NC	NC	NC	0	—	—	NM	NM	NM

RCT: randomized controlled trial; AL: allogenous graft; AT: autogenous graft; H: horizontal; V: vertical; Y: yes; N: no; MCA: mineralized cortical allograft; BBM: bobine bone mineral; NC: no clear; NM: not metioned; NCG: no control group.
